# In Vitro Cultures and Volatile Organic Compound Production in *Chiliadenus montanus* (Vhal.) Brullo

**DOI:** 10.3390/plants11101326

**Published:** 2022-05-17

**Authors:** Doaa Abu-Darwish, Rida Shibli, Ayed M. Al-Abdallat

**Affiliations:** 1 Department of Horticulture and Crop Science, School of Agriculture, The University of Jordan, Amman 11942, Jordan; doaa.abudarwish@yahoo.com (D.A.-D.); r.shibli@ju.edu.jo (R.S.); 2 Department of Agricultural Biotechnology and Genetic Engineering, Faculty of Agriculture Technology, Al-Ahliyya Amman University, Amman 19328, Jordan

**Keywords:** callus, astreraceae, headspace solid-phase micro-extraction, microshoots, micropropagation, secondary metabolites

## Abstract

Callus and microshoot cultures were established for *Chiliadenus montanus* (Vhal.) Brullo. (Asteraceae), a medicinal plant known for producing volatile organic compounds (VOCs). Callus induction was achieved successfully by culturing leaf explants on full-strength Murashige and Skoog medium (MS) supplemented with 2.2 µM 2, 4-dichlorophenoxy acetic acid (2,4-D) and 6.9 µM kinetin (Kin). Successful direct shoot regeneration was achieved using nodal explants cultured onto half-strength MS media supplemented with 1.4 μM Gibberellic Acid (GA_3_) and 4.4 μM 6-Benzylaminopurine (BAP). Indirect microshoots were successfully regenerated using callus cultured on MS media supplemented with 8.8 μM BAP, 2.2 μM Zeatin, and 1.4 μM GA_3_ followed by culturing on MS media supplemented with 8.8 μM BAP and 0.5 μM naphthalene acetic acid (NAA). Using wild plant aerial parts, callus and microshoots samples, VOCs were extracted successfully using Headspace Solid-Phase Micro-Extraction (HS-SPME) and analyzed by gas chromatography–mass spectrometry (GC-MS). In wild plant extracts, sesquiterpene hydrocarbons were found to be predominant with the following principal components: Alloaromadendrene (11.92%), trans-Cadina-1(6),4-diene (7.54%), and α-caryophyllene (6.77%). The analysis of in vitro microshoots revealed high levels of oxygenated monoterpenes with *cis*-Myrtanol (16.62%), and β-Cyclocitral (14.3%) as the main components. Callus extract was dominated by monoterpene hydrocarbons and the main compounds identified were (Z)-β-Ocimene (22.27%), p-Cymene (15.13%), and α-pinene (13.78%). In conclusion, an efficient in vitro production system of VOCs in *C. montanus* was established that can be used in the future for boosting their production without endangering wild plants.

## 1. Introduction

The family Asteraceae is one of the largest families of flowering plants and it includes over 1600 genera and 25,000 species of herbs, shrubs, and trees [1]. *Chiliadenus* is a small genus that belongs to the Asteraceae family and it consists of ten species distributed along the Mediterranean Sea [2,3]. *Chiliadenus montanus* (Vahl.) Brullo (synonymous: *Jasonia montana*, *Varthemia montana* (Vahl.) Boiss.) is an aromatic medicinal plant that is mainly distributed in rocks, steppes, and desert areas in the eastern parts of the Mediterranean region [4]. *Chiliadenus montanus* is described as an herbaceous plant, almost 50 cm in height with yellow discoid heads, hermaphrodite flowers, and hairy alternate leaves found on small lateral shoots [5]. The plant is also described as a woody perennial herb that has a strong pleasant aromatic odor and it usually blooms from September to November [6]. 

*Chiliadenus montanus* is commonly used as a traditional medicinal plant, popularly known as Haneida, and it is used in folk medicine to treat several diseases and disorders [7]. The dried leaves of *C. montanus* have been widely used by Bedouins as an herbal tea to treat renal troubles, diarrhea, stomachaches, and chest diseases [8,9]. Phytochemical studies of *C. montanus* identified monoterpenes, sesquiterpenes, diterpenes, triterpenes, sterols, and flavonoids as major compounds in its natural extracts [10,11,12,13]. The chemical composition of its essential oils showed high scavenging activity against peroxyl radicals [14]. Furthermore, the plant was found to exhibit anticancer [15,16], antioxidant [17], anticholestatic [18], and antimicrobial activities [19] as well as antiatherogenic [20] and anti-diabetic efficiency [21,22]. Therefore, *C. montanus* is considered one of the most valuable medicinal plants in the region and it has great potential as a therapeutic agent against multiple diseases [23].

The *C. montanus* plant is considered a vulnerable neglected herb that grows in marginal habitats and it is subjected to threats of extinction due to overgrazing, urbanization, and climate change [1,24]. Additionally, it is considered naturally endophytic of many fungi and bacteria, which makes it very challenging to establish a successful micropropagation system for this plant [25]. Sexual propagation of *C. montanus* was reported previously and was associated with limited seed viability and production because it only blooms for two months during the growing season [26]. To date, the establishment of in vitro tissue cultures for the *C. montanus* plant and the utilization of such systems for the production of secondary metabolites have not been reported. Hence, a micropropagation protocol for the *C. montanus* plant through in vitro techniques is considered a promising approach for its multiplication and conservation and the ex-situ production of volatile organic compounds (VOCs). 

The main objectives of this study were to establish in vitro tissue cultures for *C. montanus* using different explant types, media compositions, and growth conditions and to identify VOCs produced from *C. montanus* callus and microshoot cultures. Moreover, in vitro cultures of *C. montanus* were evaluated for their VOCs contents and compositions using the headspace solid-phase microextraction (HS-SPME) technique coupled with gas chromatography-mass spectrometry (GC-MS) analysis.

## 2. Materials and Methods

### 2.1. Plant Material

**Figure 1:**
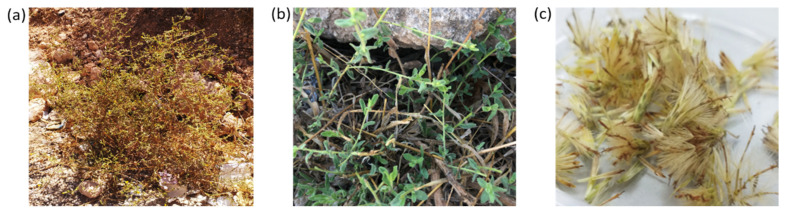
(**a**) *C. montanus* (Vahl.) Brullo plant growing in the wild; (**b**) close view of *C. montanus* shoots in the wild; and (**c**) *C. montanus* seeds collected from the wild plant.

A *C. montanus* wild plant was identified in the Al-Kamsheh region in Zarqa governorate, Jordan (32°07′28.4″ N; 35°53′22.1″ E) (Figure 1). The whole plant was transplanted into a large pot (10 L in volume) containing a mix of soil and peat moss (1:1) and kept under greenhouse conditions with proper management at the School of Agriculture/The University of Jordan. From this plant, different explant types were used for the establishment of in vitro cultures that were analyzed for the production of VOCs using HS-SPME and GC-MS techniques.

### 2.2. Callus Culture Establishment

For the establishment of callus culture, leaves of *C. montanus* were excised from greenhouse plants and used as explants. Initially, the explants were washed under running tap water to remove dirt particles and then treated with 70% (*v*/*v*) ethanol for 30 seconds under aseptic conditions before washing thoroughly three times with sterilized distilled water. The explants were then washed with 1% (*v*/*v*) sodium hypochlorite solution containing a few drops of Tween^®^ 20 for one minute and then rinsed six times with sterilized distilled water and dried on sterile paper before their use. For callus induction, the disinfected explants were cultured on Murashige and Skoog (MS) media [27] supplemented with 0.1 M sucrose, 100 mg·L^−1^ myo-inositol, and different concentrations of either naphthalene acetic acid (NAA) (2.6, 5.2, 8.0, and 10.7 µM) or 2,4-dichlorophenoxy acetic acid (2,4-D) (2.2, 4.5, 6.7, and 9.0 µM) as auxin sources in combination with either 6-Benzylaminopurine (BAP) (2.2, 4.4, 6.6, and 8.8 µM) or kinetin (Kin) (2.3, 4.6, 6.9, and 9.2 µM) as cytokinin sources and the pH of the media was adjusted to 5.8 before adding 6 g/lagar (Bacto-agar, Difco, India) as described previously [28]. The media was autoclaved for 15 min at 121 °C and 1.06 kg·cm^−2^ and 25 mL was dispensed into Petri dishes. The disinfected explants were placed horizontally on the media and incubated for eight weeks at 25 ± 2 °C under complete darkness. 

A Complete Randomized Design (CRD) was used and each treatment was replicated five times (each replicate consisted of two explants in a single Petri dish), and the cultures monitored for callus development. At the end of the incubation period, data were collected for callus diameter, fresh weight, texture, and color. Data were analyzed using SAS (version 9, SAS Inc., Cary, NC, USA); an analysis of variance (ANOVA) was obtained for each experiment, and mean separation at a probability level of 0.05 was performed according to Tukey’s HSD test. The source of all growth regulators used in this study was Sigma-Aldrich Chemical Co., St. Louis, MO, USA. The best callus induction media was used for callus maintenance that was based on the best callus growth, in terms of increase in fresh weight and morphological changes, and was named thereafter as the callus maintenance medium with a subculture event every four weeks.

### 2.3. Microshoot Cultures Establishment

For the direct regeneration of *C. montanus* microshoots, nodal segments were excised from in vivo shoots and used as explants. For this purpose, shoots were cut into small segments (3 cm in length) that contained two buds per segment. The nodal explants were then sterilized as described above. The disinfected explants were then cultured on different media including full-strength MS media supplemented with 0.1 M sucrose and 100 mg·L^−1^ myo-inositol, half-strength White media [29] supplemented with 0.05 M sucrose and 100 mg·L^−1^ myo-inositol and half-strength MS media supplemented with 0.05 M sucrose, 100 mg·L^−1^ myo-inositol and 4.4 µM BAP in combination with 1.4 µM Gibberellic Acid (GA_3_). The explants were placed vertically inside an Erlenmeyer flask (250 mL) containing 150 mL of media and then incubated for four weeks at 25 ± 2 °C under a 16 h photoperiod and irradiance of 56 μmol·m^−2^·s^−1^ provided by cool white fluorescent lamps (Philips, Germany). Each treatment was replicated 10 times (each replicate was represented by one explant in a flask) and at the end of the incubation period, data were collected for the number of leaves per shoot and final shoot length.

For rooting, microshoots (~1 cm length) regenerated directly from nodal explants were cut off and transferred into half-strength MS media supplemented with different concentrations of auxins: indole acetic acid (IAA) (2.2, 4.5, 6.8, and 11.4 µM), NAA (2.1, 4.2, 6.4, and 10.7 µM), or indole butyric acid (IBA) (1.9, 3.9, 5.9, and 9.8 µM). The treatments were arranged in CRD with 10 replicates per treatment with one microshoot per replicate. The percentage of root induction, root numbers, and the growth state of the roots were recorded after four weeks of culturing.

For the establishment of microshoot cultures using callus pieces as explant, 0.5 g of callus was cultured on MS media supplemented with 0.1 M sucrose, 100 mg·L^−1^ myo-inositol, and a combination of 8.8 μM BAP, 2.2 μM Zeatin, and 1.4 μM GA_3_. In vitro microshoots were regenerated from calli after four weeks of culture and the microshoots were allowed to grow until they reached a length of one cm. Thereafter, individual microshoots were excised and cultured on MS media supplemented with 0.1 M sucrose and 100 mg·L^−1^ myo-inositol and different levels of BAP (9.8, 19.7, and 29.5 μM) or Kin (9.2, 18.5, and 27.8 μM) in combination with 0.5 μM NAA and the cultures were incubated for another four weeks at 25 ± 2 °C under a 16 h photoperiod. A CRD was used, and each treatment was replicated 10 times (each replicate was represented by a single microshoot), and at the end of the incubation period, data were collected for the number of leaves per shoot and shoot length. Data were analyzed statistically as described in callus culture experiments.

### 2.4. Volatile Organic Compound Extraction and Analysis

Three samples of *C. montanus* were used for VOC analysis that included shoots (including aerial parts: leaves and stem) collected in September 2020 from the wild plant, one-month-old microshoots that were grown on half-strength MS media supplemented with 0.1 M sucrose, mg·L^−^^1^ myo-inositol, 4.4 µM BAP in combination with 1.4 µM GA_3_ and 0.5 µM NAA, and calli that were grown on callus maintenance medium under complete darkness for one month. Volatile organic compounds were extracted from the three samples by using the headspace solid-phase microextraction (HS-SPME) technique coupled with gas chromatography-mass spectrometry (GC-MS), as described previously [30,31] with some modifications. For this purpose, the plant samples were air-dried at room temperature in the shade until they reached a constant weight. The dried samples were powdered, and 0.3 g were extracted three times with 0.5 mL *n*-hexane (Scharlau, Barcelona, Spain) with sonication (Bandelin Sonorex, Bandelin electronics, Germany) for 10 min. The extracts were then filtered and dried with sodium sulfate anhydrous (Na_2_SO_4_) (UCB, Bruxelles, Belgium) and then transferred to a 10 mL uncapped headspace glass vial (Supelco, Cassopolis, MI, USA) and left for 30 min to fully evaporate the *n*-hexane. Thereafter, the vials were capped and incubated at 50.0 °C for 0.5 min, where a 1 cm 50/30 µm DVB/CAR/PDMS coated SPME fiber (Supelco, Cassopolis, MI, USA) was exposed to the headspace for 30 min to achieve equilibrium. Thereafter, the VOCs were desorbed from the SPME fiber and GC-MS analyses were carried out using Varian chrompack CP-3800 C/MS/MS-200 (Saturn, The Netherlands) fitted with a DP-5 (5% diphenyl, 95% dimethyl polysiloxane) GC capillary column (30 m × 0.25 mm × 0.25 μm film thicknesses) as described previously [32]. Peak identifications with specific retention time was done using a mixture of (C8–C20) alkanes in hexane (Sigma Aldrich, Saint Louis, MI, USA) that was injected in the same condition already described for GC-MS analysis, and retention indexes were calculated [33]. The VOCs of the plant samples were identified by comparing the mass spectra of each peak with those reported in the mass spectral database (National Institute of Standards and Technology, Standard Reference Data Program, Gaithersburg, MD 2089, USA). The relative content of each identified compound was calculated as the peak area of the total area of all the identified peaks, and a standard curve was used to calculate the area under the peak. Each plant sample was analyzed by HS-SPME and GC-MS in three replicates.

## 3. Results and Discussion

### 3.1. Callus Culture

**Figure 2:**
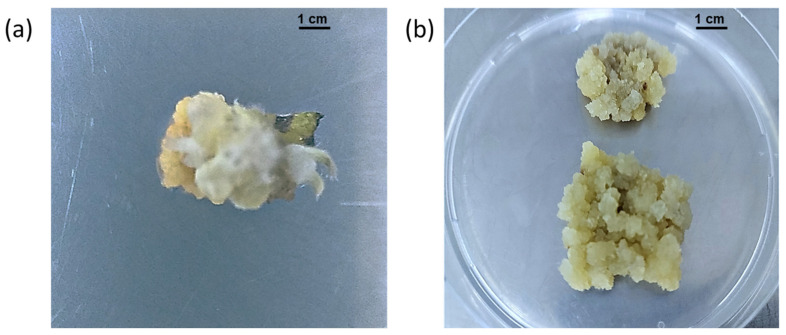
Callus induction using a leaf explant of *C. montanus* on MS media supplemented with 2.2 µM 2,4-D and 6.9 µM Kin (**a**) Callus induction after four weeks (**b**) Four-week-old callus culture growing on callus maintenance media.

Weak callus induction was observed on MS media lacking plant growth regulators and clear variations were observed between tested media types supplemented with different combinations of auxin and cytokinin types and concentrations (Table S1). Murashige and Skoog media supplemented with 2.2 µM 2,4-D and 6.9 µM Kin produced, significantly, the highest fresh weight (2 g) and callus diameter (2.82 cm) with the best callus texture and color (Figure 2), followed by MS media supplemented with 9.0 µM 2,4-D and 2.3 µM Kin, which produced compact and yellow-brown callus with a fresh weight of 1.608 g and a callus diameter of 2.46 cm. For the rest of the callus induction treatments, weak callus growth was observed with calli being pale brown, green, and compact in texture (Table S1). The MS media supplemented with 2.2 µM 2,4-D and 6.9 µM Kin was used for callus maintenance by sub-culturing 250 mg of callus in a Petri dish every four weeks (Figure 2b). To the best of our knowledge, this is the first report on the establishment of a callus culture in *C. montanus*. Callus induction in *Ruta graveolens* L. was successfully achieved on MS media supplemented with 5 µM 2,4-D and 7 µM Kin [34] and the combination of 2,4-D and Kin was essential to induce callus in the *Elephantopus scaber* plant that belongs to the Asteraceae family [35]. 

### 3.2. Microshoots Culture

**Figure 3:**
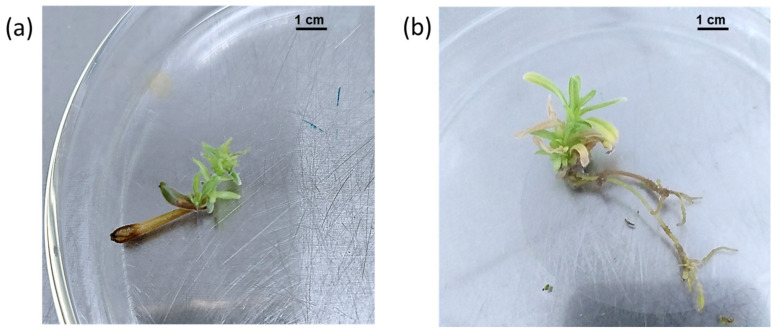
(**a**) Effect of half-strength MS medium supplemented with 4.4 µM BAP and 1.4 µM GA_3_ on *C. montanus* microshoot growth after four weeks of incubation; (**b**) Effect of half-strength MS medium supplemented with 3.9 µM IBA on in vitro rooting of *C. montanus* after four weeks of incubation.

In this study, direct shoot regeneration from axillary buds on nodal segments cultured on half-strength MS medium supplemented with 0.05 M sucrose, 4.4 µM BAP, and 1.4 µM GA_3_ proved to be superior when compared to half-strength White media and hormone-free MS media (Table 1; Figure 3a). This media produced the highest mean value for shoot length (1.51 cm) and the highest mean value for the number of leaves (13.8). On the other hand, all nodal segments cultured on half-strength White media produced shorter shoots (0.56 cm) with a lower number of leaves (5.1). Nodal segments cultured on hormone-free MS media showed no bud breakage and no microshoots were produced (Table 1). In general agreement with the result of this study, MS media supplemented with 4.4 μM BAP, 4.6 μM Kin, or 4.9 μM 2-isopentenyladenine (2ip) in combination with 1.4 μM GA_3_ produced the maximum number of shoots in *Eclipta alba* [36]. Half-strength MS medium supplemented with 4.4 μM BAP and 1.4 μM GA_3_ was more efficient in shoot culture establishment of *Baccharis antioquensis*, a member of the Asteraceae family, than hormone-free MS media [37]. On the other hand, using White media for culturing *Centaurea zeybekii* shoots resulted in slower shoot growth and a low transplantation rate when compared to half-strength MS media [38]. The superiority of half-strength MS medium may reflect the requirement of a relatively low concentration of salts for the growth of *C. montanus* shoots, which is in general agreement with the results of [39] who indicated that low-ion strength MS medium resulted in the high growth of shoots and roots of *Stevia rebaudiana* Bertoni, compared to a full-strength medium. 

For the rooting experiment, from all tested treatments, only three microshoots produced roots; one of them was obtained on half-strength MS media supplemented with 0.05 M sucrose and 4.2 µM NAA (10%), another on MS media supplemented with 10.7 µM NAA (10%), and the last one on MS media supplemented with 3.9 µM IBA (10%) (Figure 3b). Similar results were obtained by [40] where rooting of *Echinacea* spp. was unsuccessful for most of the treatments, except for 4 µM NAA. 

**Figure 4:**
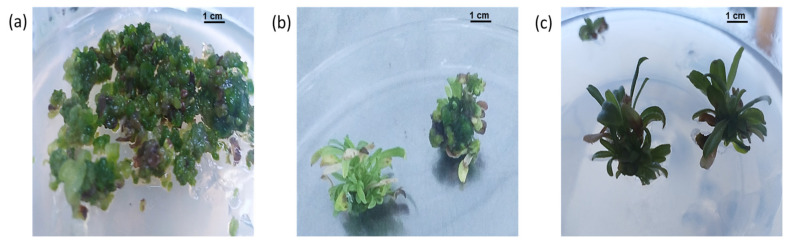
(**a**) *C. montanus* microshoots induction on MS medium supplemented with 8.8 μM BAP, 2.2 μM Zeatin, and 1.4 μM GA_3_*;* (**b**) Microshoots after four weeks of culture on MS medium supplemented with 8.8 μM BAP, 2.2 μM Zeatin, and 1.4 μM GA_3_; (**c**) Microshoots growth on MS medium supplemented with 8.8 μM BAP and 0.5 μM NAA after four weeks of culture.

For indirect shoot regeneration using callus as an explant, successful regeneration was achieved using MS media supplemented with 0.1 sucrose and a combination of 8.8 μM BAP, 2.2 μM Zeatin, and 1.4 μM GA_3_ (Figure 4a,b). Microshoots were allowed to grow until they reached one cm before sub-culturing on MS medium supplemented with 0.1 M sucrose and different levels of BAP (8.8, 17.7, and 26.6 μM) or Kin (9.2, 18.5, and 27.8 μM) in combination with 0.5 μM NAA. The shoot length and number of leaves of microshoots were significantly affected by cytokinin type and concentrations (Table 2). The highest mean values for shoot length (2.25 cm) and number of leaves (19.70) were obtained from microshoots cultured on MS medium supplemented with 8.8 μM BAP and 0.5 μM NAA, which was significantly different when compared to other treatments (Figure 4c; Table 2).

### 3.3. Volatile Organic Compound Analysis

The VOC components in wild, microshoot, and callus extracts of *C. montanus* were identified using GC-MS analysis based on the comparison of the obtained RI and MS fragmentation patterns to standard compounds and computer matching with built-in libraries [33]. Accordingly, the wild plant extract was found to be richer in sesquiterpene hydrocarbons (47.97%) and oxygenated sesquiterpenes (35.32%), followed by oxygenated monoterpenes (12.22%) and monoterpene hydrocarbons (1.68%) (Table 3). On the other hand, the most abundant chemical groups in microshoot extracts were oxygenated monoterpenes (58.09%) and monoterpene hydrocarbons (23.68%), followed by sesquiterpene hydrocarbon (10.16%) and oxygenated sesquiterpenes (1.14%), while callus extracts contained only monoterpene hydrocarbons (63.35%) and oxygenated monoterpenes (35.64%) (Table 3). The occurrence of monoterpenes and sesquiterpenes in *C. montanus* was reported previously [15,41]. Ref. [41] reported that the predominant compounds identified in *C. montanus* wild-growing plants were oxygenated monoterpenes, which was not in general agreement with [15]. In another study, clear variations in the composition of VOCs between wild populations of *C. iphionoides* were also observed [42]. In this study, microshoot and callus extracts produced higher amounts of monoterpenes when compared with the wild-type plant extract, which produced mainly sesquiterpenes compounds. In contrast to microshoot and wild plant extracts, callus culture extracts produced only monoterpenes compounds; such differences in VOC profiles between in vitro cultures and wild plant extracts were reported previously [43].

The analysis of VOCs obtained from the areal parts of wild *C. montanus* resulted in the identification of 19 compounds representing 97.19% of the total oil content. The main VOCs found in wild plant extract included Alloaromadendrene (11.92%), trans-Cadina-1(6),4-diene (7.54%), and α-caryophyllene (6.77%) from the sesquiterpene hydrocarbons group, while from the oxygenated sesquiterpenes group, Muurola-4,10(14)-dien-8.α-ol (22.19%) and δ-amorphene (9.32%) were the most abundant compounds (Table 4). For the monoterpenoids group, Thymol methyl ether (9.17%) and γ-Terpineol (3.05%) were detected in wild plant extract (Table 4). In the microshoot extracts, 26 compounds were identified that accounted for 99.22% of the total VOCs with cis-Myrtanol (16.62%) and β-Cyclocitral (14.3%) as the main compounds from the oxygenated monoterpenes group, and p-cymene (8.25%), (+)-2-Carene (6.43%), and (E)*-*β-ocimene as the main compounds from the monoterpene hydrocarbons group (Table 4). For sesquiterpenes in microshoot extracts, α-caryophyllene (2.80%) and δ-Amorphene (2.35%) were identified as the main components. For callus extracts, the VOCs profile was dominated by monoterpenes, and 15 compounds were identified, representing 98.99% of the total content (Table 4). The main compounds of this fraction were (Z)-β-Ocimene (22.27%), p-Cymene (15.13%), and α-Pinene (13.78%) from the monoterpene hydrocarbons group, while Thymoquinone was as the main compound in the oxygenated monoterpenes group (13.25%). 

The composition of the microshoot, wild, and callus VOCs showed qualitative and quantitative differences. For instance, the number of different constituents identified in microshoot extract was higher compared to the wild plant and in vitro callus, which produced the lowest number of compounds (Table 4). In addition, fluctuations in the concentration of common compounds were observed, where, for instance, α-Caryophyllene was higher in wild plant extract (6.77%) when compared to microshoot extract (1.73%). Ref. [15] identified α-Caryophyllene as a major compound in the VOCs of *C. montanus* from the Sinai region in Egypt and this is in general agreement with the findings of this study. Interestingly, *p*-Cymene was identified only in callus and microshoot extracts, although it was previously identified in wild *C. montanus* plants [44] and other related species [42,43,44,45,46,47,48]. In general, several identified compounds in this study were reported previously in wild *Chiliadenus spp*. from the region. For instance, α-Caryophyllene was detected previously in *C. iphionoides* [46], *C. lopadusanus* [47], and *C. antiatlanticus* [45]. Ref. [45] reported 15.5% α-pinene in the aerial parts of *C. antiatlanticus,* while in this study, 13.78% were detected in *C. montanus* callus extract. They also detected γ-Terpinene (0.8%) that was identified in the *C. montanus* microshoot and callus extracts (0.74% and 1.53%, respectively) of this study. Finally, the qualitative and quantitative variations in VOCs between intact plants and in vitro cultures were reported previously in *Artemisia spicigera* in vitro cultures [43] which is in general agreement with the results of this study. Such variations in VOCs between *C. montanus* samples used in this study could be attributed to environmental factors affecting their production in natural habitats such as light, temperature, water, and nutrients. For most plants, while under in vitro conditions, such factors are controlled continuously and adjusting any individual factor will result in altering their levels [48]. 

## 4. Conclusions

In this study, efficient micropropagation and in vitro culture systems for *C. montanus* were successfully established using different types of explants and media compositions. Callus culture was successfully established using leaf explants cultured on full-strength MS media supplemented with 2.2 µM 2,4-D and 6.9 µM kinetin Kin. Microshoots were successfully regenerated directly from nodal explants cultured onto half-strength MS media supplemented with 1.4 μM GA_3_ and 4.4 μM BAP, while indirect microshoots were regenerated using callus explant cultured on MS media supplemented with 8.8 μM BAP, 2.2 μM Zeatin, and 1.4 μM GA_3_. The VOCs from in vivo and in vitro samples were extracted successfully using HS-SPME and analyzed using GC-MS. Clear variations in the qualitative and quantitative aspects of VOCs between the wild plant and in vitro cultures of *C. montanus* were observed. Callus cultures were found to be rich in monoterpene hydrocarbons, while microshoot cultures were rich in oxygenated monoterpenes when compared to the wild plant extract that was rich in sesquiterpenes. In conclusion, the in vitro cultures of *C. montanus* reported in this study can be used to boost the production of VOCs under aseptic conditions without affecting wild-grown plants.

## Figures and Tables

**Table 1:** Effect of different media types on direct shoot bud initiation from nodal explants (n = 10) after four weeks of in vitro culture of *C. montanus*.

Type of Media	Number of Leaves	Shoot Length (cm)
1/2 MS media supplemented with 1.4 μM GA_3_ + 4.4 μM BAP	13.80 ± 0.36 ^a*^	1.51 ± 0.024 ^a^
Half strength White media	5.10 ± 0.36 ^b^	0.56 ± 0.024 ^b^
Hormone-free and full-strength MS media	0.00 ± 0.36 ^c^	0.00 ± 0.024 ^c^

^*^ Mean values with different letters are significantly different according to Tukey’s HSD test at *p* < 0.05 and the standard error of means.

**Table 2:** Effect of different media types on microshoots growth after four weeks of incubating four-week-old microshoots (n = 10) regenerated from callus culture of *C. montanus*.

Cytokinin Type	Concentration (μM)	Number of Leaves	Shoot Length (cm)
BAP	0.0	10.70 ± 0.74 ^c^*	1.09 ± 0.09 ^c^
	8.8	19.70 ± 0.74 ^a^	2.25 ± 0.09 ^a^
	17.7	15.00 ± 0.74 ^d^	1.57 ± 0.09 ^d^
	26.6	7.50 ± 0.74 ^g^	0.80 ± 0.09 ^g^
Kin	0.0	10.00 ± 0.74 ^e^	1.01 ± 0.09 ^e^
	9.2	17.60 ± 0.74 ^b^	2.00 ± 0.09 ^b^
	18.5	9.30 ± 0.74 ^f^	0.90 ± 0.09 ^f^
	27.8	5.00 ± 0.74 ^h^	0.56 ± 0.09 ^h^

^*^ Mean values with different letters are significantly different according to Tukey’s HSD test at *p* < 0.05 and the standard error of means.

**Table 3:** Classification and content (%) of VOCs identified by GC-MS analysis in different extracts of *C. montanus*.

	Content (%)		
Chemical Classes	Whole Wild Plant	Microshoots	Callus Culture
Total Monoterpenes	13.9	81.77	98.99
Oxygenated monoterpenes	12.22	58.09	35.64
Monoterpene hydrocarbons	1.68	23.68	63.35
Total Sesquiterpenes	83.29	11.30	-
Oxygenated sesquiterpenes	35.32	1.14	-
Sesquiterpene hydrocarbons	47.97	10.16	-
Total Identified	97.19	99.22	98.99
Not-identified	2.81	0.78	1.01

**Table 4:** Volatile organic compounds (VOCs) identified in different sample extracts of *C. montanus* and their content.

				Content (%) ^1^		
Compound Name	Classification	RI- Exp ^2^	RI-Lit ^3^	Wild	Microshoot	Callus
α-Pinene	Monoterpene hydrocarbons	946	939	-	-	13.78
α-Phellandrene	Monoterpene hydrocarbons	1007	1002	-	-	3.56
(+)-2-Carene	Monoterpene hydrocarbons	1011	1002	-	6.43	-
p-Cymene	Monoterpene hydrocarbons	1033	1024	-	8.25	15.13
(Z)- β-Ocimene	Monoterpene hydrocarbons	1039	1037	-	1.23	22.27
(*E*)- β- Ocimene	Monoterpene hydrocarbons	1054	1050	-	6.30	-
γ-Terpinene	Monoterpene hydrocarbons	1066	1059	-	0.74	1.53
Cis-Sabinene hydrate (lPP vs. OH)	Monoterpene hydrocarbons	1079	1070	-	0.73	4.74
6-Camphenolle	Oxygenated Monoterpenes	1105	1096	-	1.00	-
Linalool	Oxygenated Monoterpenes	1109	1096	-	0.88	-
Cis-Thujone	Oxygenated Monoterpenes	1111	1102	-	-	2.56
Trans-Thujone	Oxygenated Monoterpenes	1116	1114	-	-	3.24
1-Undecyne	Monoterpene hydrocarbons	1130	1125	-	-	6.45
α-Terpineol	Monoterpene hydrocarbons	1136	1133	-	1.50	-
(Z)-Tagetone	Oxygenated monoterpenes	1159	1152	-	-	2.10
1,4-Dimethoxybenzene	Non-terpenoid hydrocarbon	1165	1165	-	6.15	-
Pyrazine, 2-mcthoxy-3-(1-methylpropyl)-	Monoterpene hydrocarbons	1179	1172	-	3.5	-
trans-1(7),8-p-Menthadien-2-ol	Oxygenated monoterpenes	1189	1189	-	1.58	-
o-Cumenol	Oxygenated monoterpenes	1203	1196	-	2.27	-
γ-Terpineol	Oxygenated monoterpenes	1204	1199	3.05	-	1.51
β-Cyclocitral	Oxygenated monoterpenes	1227	1219	-	14.3	-
(-) –Neoisodihydrocarveol	Oxygenated monoterpenes	1234	1228	-	0.84	-
Thymol, methyl ether	Oxygenated monoterpenes	1237	1235	9.17	-	-
Thymoquinone	Oxygenated monoterpenes	1257	1252	-	-	13.25
cis-Myrtanol	Oxygenated monoterpenes	1258	1253	-	16.62	-
Geranial	Monoterpene hydrocarbons	1274	1267	-	0.92	-
Isopulegyl acetate	Oxygenated monoterpenes	1282	1277	-	1.62	-
neo-is-3-*Thujyl* acetate	Oxygenated monoterpenes	1289	1283	-	2.79	-
Azulene	Monoterpene hydrocarbons	1308	1298	1.68	-	-
neo-dihydro carveol acetate	Oxygenated monoterpenes	1365	1359	-	-	3.81
Dihydro eugenol	Oxygenated monoterpenes	1375	1369	-	6.51	-
α-Copaene	Sesquiterpene hydrocarbons	1381	1376	4.25	-	-
Geranyl acetate	Oxygenated monoterpenes	1383	1381	-	3.76	-
Isobornyl propionate	Oxygenated monoterpenes	1384	1384	-	-	4.43
β-Caryophyllene	Sesquiterpene hydrocarbons	1425	1419	5.15	-	-
β-Copaene	Sesquiterpene hydrocarbons	1436	1432	0.56	-	-
α-caryophyllene	Sesquiterpene hydrocarbons	1462	1454	6.77	4.53	-
Alloaromadendrene	Sesquiterpene hydrocarbons	1467	1460	11.92	-	-
β-Acoradiene	Sesquiterpene hydrocarbons	1475	1470	1.59	1.95	-
trans-Cadina-1(6),4-diene	Sesquiterpene hydrocarbons	1480	1476	7.54	-	-
α-Zingiberene	Sesquiterpene hydrocarbons	1493	1493	1.01	-	-
γ-Amorphene	Oxygenated sesquiterpenes	1503	1495	4.35	-	-
Premnaspirodiene	Sesquiterpene hydrocarbons	1512	1506	0.61	-	-
δ-*amorphene*	Sesquiterpene hydrocarbons	1519	1512	9.32	2.35	-
γ-Cadinene	Sesquiterpene hydrocarbons	1521	1513	-	1.33	-
7-epi-*α*-Selinene	Sesquiterpene hydrocarbons	1523	1522	1.70	-	-
trans-Calamenene	Sesquiterpene hydrocarbons	1528	1522	2.52	-	-
Spathulenol	Oxygenated sesquiterpenes	1578	1578	2.22	-	-
Gleenol	Oxygenated sesquiterpenes	1589	1587	1.59	-	-
Muurola-4,10(14)-dien-8. α.-ol	Oxygenated sesquiterpenes	1634	1631	22.19	-	-
epi-*α*-Cadinol	Sesquiterpene hydrocarbons	1649	1640	-	1.14	-

^1^ The contents percentage is based on the GC peak; ^2^ RI-Exp: experimental retention index related to (C8–C20) n-alkanes; ^3^ RI-Lit: reported retention index.

## Data Availability

The data sets supporting the results of this article will be freely available upon reasonable request from A.M.A.

## Supplementary Materials

The following supporting information can be downloaded at: https://www.mdpi.com/article/10.3390/plants11101326/s1, Table S1: Effect of different concentrations of auxins and cytokinins on callus induction using leaf explants of *C. montanus*.
