# Supplementary material for: In Vitro Cultures and Volatile Organic Compound Production in Chiliadenus montanus (Vhal.) Brullo
Source: Plants (Basel). 2022 May 17;11(10):1326. doi: 10.3390/plants11101326 (PMC9148159; doi:10.3390/plants11101326)
Supplement: Supplementary file 1 [file plants-11-01326-s001.zip › plants-1690538-supplementary.pdf]

Table S1. Effect of different concentrations of auxins and cytokinins on callus induction using leaf explants of *C. montanus*.

Treatment	Callus weight (g)	Callus diameter (cm)	Callus color	Callus texture
MS free of hormones	0.10 u	0.36 s	Brown	Compact
2.3 µM Kin	0.15 tu	0.48 qrs	Yellow	Compact
4.6 µM Kin	0.284 p-u	0.62 m-s	Yellow	Compact
6.9 µM Kin	0.316 o-u	0.68 l-s	Yellow+Brown	Compact
9.2 µM Kin	0.424 m-s	0.74 k-r	Yellow+Brown	Embryonic callus
4.5 µM 2,4-D + 4.6 µM Kin	1.200 d	1.62 de	Yellow+Green	Compact
4.5 µM 2,4-D + 6.9 µM Kin	0.288 p-u	0.60 n-s	Yellow	Friable
6.7 µM 2,4-D	0.498 k-p	0.80 h-o	Yellow+Brown	Root + callus
6.7 µM 2,4-D + 4.6 µM Kin	1.384 cd	1.90 cd	Green+Brown	Compact
9.0 µM 2,4-D	0.556 i-n	0.86 h-o	Brown	Friable
9.0 µM 2,4-D + 2.3 µM Kin	1.608 b	2.46 b	Yellow+Brown	Friable
9.0 µM 2,4-D + 6.9 µM Kin	0.458 l-r	0.76 k-r	Yellow+Brown	Compact
2.2 µM 2,4-D	0.460 l-r	0.74 k-r	Yellow	Compact
2.2 2,4-D + Kin 2.3	0.728 f-j	1.12 g-j	Yellow+brown	Compact
2.2 µM 2,4-D + 4.6 µM Kin	0.228 stu	0.44 rs	Green	Compact
2.2 µM 2,4-D + 6.9 µM Kin	2.084 a	2.82 a	Yellow	Friable
2.2 µM 2,4-D + 9.2 µM Kin	1.438 bc	2.10 c	Brown+Green	Compact
4.5 µM 2,4-D	0.216 stu	0.46 rs	Yellow+Brown	Root + callus
4.5 µM 2,4-D + 2.3 µM Kin	0.964 e	1.46 ef	Yellow	Friable
4.5 µM 2,4-D + 9.2 µM Kin	0.914 ef	1.36 efg	Green+Brown	Compact
6.7 µM 2,4-D + 2.3 µM Kin	0.718 f-k	1.02 h-k	Yellow+Brown	Friable
6.7 µM 2,4-D + 6.9 µM Kin	0.374 m-s	0.72 k-r	Green	Compact
6.7 µM 2,4-D + 9.2 µM Kin	0.284 p-u	0.54 o-s	Yellow+Green	Compact
9.0 µM 2,4-D + 4.6 µM Kin	0.352 n-t	0.64 m-s	Yellow+Brown	Compact
9.0 µM 2,4-D + 9.2 µM Kin	0.324 o-u	0.64 m-s	Yellow+Brown	Compact
2.2 µM BAP	0.214 stu	0.54 o-s	Yellow+Green	Friable
4.4 µM BAP	0.392 m-s	0.70 k-r	Yellow+Green	Compact
6.6 µM BAP	0.538 j-o	0.82 i-p	Yellow+Grown	Compact
8.8 µM BAP	0.596 h-m	0.90 h-n	Yellow+Brown	Compact
2.6 µM NAA	0.480 l-q	0.76 k-r	Yellow+Green	Compact

5.2 µM NAA + 4.4 µM BAP	0.254 r-u	0.54 o-s	Yellow+Green	Compact
5.2 µM NAA + 6.6 µM BAP	0.288 p-u	0.54 o-s	Yellow	Friable
8.0 µM NAA	0.278p-u	0.54 o-s	Yellow	Root + callus
8.0 µM NAA + 4.4 µM BAP	0.272 q-u	0.50 p-s	Yellow	Friable
10.7 µM NAA	0.664 g-l	0.94 h-m	Yellow	Root + callus
10.7 µM NAA + 2.2 µM BAP	0.786 e-h	1.14 f-i	Yellow	Friable
10.7 µMNAA + 6.6 µM BAP	0.678 g-l	0.98 h-l	Yellow+Brown	Compact
2.6 µM NAA	0.264 q-u	0.54 o-s	Yellow	Compact
2.6 µM NAA + 2.2 µM BAP	0.294 p-u	0.56 o-s	Yellow+Green	Compact
2.6 µM NAA + 4.4 µM BAP	0.256 r-u	0.54 o-s	Green	Compact
2.6 µM NAA + 6.6 µM BAP	0.732 f-j	1.18 fgh	Yellow	Friable
2.6 µM NAA + 8.8 µM BAP	0.778 e-i	1.10 g-j	Yellow	Friable
5.2 µM NAA	0.830 efg	1.16 fgh	Yellow	Root + callus
5.2 µM NAA + 2.2 µM BAP	0.210 stu	0.48 qrs	Yellow	Friable
5.2 µM NAA + 8.8 µM BAP	0.312 p-u	0.54 o-s	Yellow+Brown	Compact
8.0 µM NAA + 2.2 µM BAP	0.268 q-u	0.54 o-s	Yellow	Friable
8.0 µM NAA + 6.6 µM BAP	0.280 p-u	0.54 o-s	Yellow+Brown	Compact
8.0 µM NAA + 8.8 µM BAP	0.818 e-h	1.16 fgh	Yellow+green	Compact
10.7 µM NAA + 4.4 µM BAP	0.710 f-k	0.98 h-l	Yellow+Brown	Friable
10.7 µM NAA + 8.8 µM BAP	0.720 f-k	1.00 h-l	Yellow+Brown	Compact

[*] **Mean values with different letters are significantly different according to Tukey's HSD test at** $p < 0.05$.
